# High–temperature droplet epitaxy of symmetric GaAs/AlGaAs quantum dots

**DOI:** 10.1038/s41598-020-62248-9

**Published:** 2020-04-16

**Authors:** Sergio Bietti, Francesco Basso Basset, Artur Tuktamyshev, Emiliano Bonera, Alexey Fedorov, Stefano Sanguinetti

**Affiliations:** 1 L–NESS and Dipartimento di Scienza dei Materiali, Università di Milano-Bicocca, via Cozzi 53, I-20125 Milano, Italy; 2 CNR–IFN and L–NESS, via Anzani 42, I–22100 Como, Italy; 3 Present Address: Now at Dipartimento di Fisica, Sapienza Università di Roma, Piazzale A. Moro 5, I-00185 Roma, Italy

**Keywords:** Quantum dots, Single photons and quantum effects, Semiconductors

## Abstract

We introduce a high–temperature droplet epitaxy procedure, based on the control of the arsenization dynamics of nanoscale droplets of liquid Ga on GaAs(111)A surfaces. The use of high temperatures for the self-assembly of droplet epitaxy quantum dots solves major issues related to material defects, introduced during the droplet epitaxy fabrication process, which limited its use for single and entangled photon sources for quantum photonics applications. We identify the region in the parameter space which allows quantum dots to self–assemble with the desired emission wavelength and highly symmetric shape while maintaining a high optical quality. The role of the growth parameters during the droplet arsenization is discussed and modeled.

## Introduction

The fabrication of high purity single and entangled photon sources is crucial for the development of quantum communication protocols^1,2^ and quantum computation^3,4^, and it is a fundamental requirement for the realization of repeaters capable of transferring quantum entanglement over long distances^5,6^. Among the different light emitting platforms, semiconductor quantum dots (QDs) are very attractive, as they can be integrated with other photonic and electronic components in miniaturized chips. Single photon and entangled photon emitters have been fabricated by QDs using self-assembly techniques like Stranski-Krastanov and Droplet Epitaxy (DE)^7^. In particular, DE enables a fine tuning of the shape, size, density, and thus, of the emission wavelength of the nanostructures^8–11^ with an emission range extending from 700 nm to 1.5 *μ*m^12^.

The high symmetry (111) surface, due to its C_3*v*_ symmetry, is optimal to reduce the fine structure splitting (FSS)^13–15^, but problematic for the growth via Stranski-Krastanov growth mode, since on (111) the relaxation of a strained III-V semiconductor epilayer immediately proceeds through the nucleation of misfit dislocation at the interface rather than through the formation of coherent 3D islands^16^. DE is able to self–assemble highly symmetric QDs on (111)A substrate, capable of polarization-entangled photon emission with very high fidelity^17^. Moreover, the choice of GaAs QDs allows a fast radiative recombination and a weaker impact of spin dephasing mechanisms^18–21^.

DE QDs have been demonstrated^22^ to improve the yield of entanglement–ready photon sources up to 95% while matching the emission wavelength with an atomic-based optical slow medium such as Rb atoms (as proposed in^23^) for the fabrication of quantum memories and quantum repeaters. This result was achieved through the combination of low values of the excitonic FSS and radiative lifetime, together with the reduced exciton dephasing allowed by the choice of GaAs/AlGaAs QDs fabricated on (111)A substrates. The major DE drawback, related to the low temperature kept for the nanostructure crystallization and barrier layer deposition necessary for this growth technique, was overcome using an innovative high–temperature DE technique which is allowed by the use of (111) substrates.

Here we present and investigate in the details a novel high–temperature DE growth technique, which is based on the control of the growth parameters (substrate temperature and As flux) on the Ga adatom diffusion during the GaAs QDs formation by droplet epitaxy on (111)A. In particular, we address the effect of Ga droplet arsenization for the formation of QDs at substrate temperature increased by about 300 °C with respect to previous reports^24^ while avoiding QD elongation^25^ due to anisotropy in Ga adatom diffusion^26–28^. We also address shape control issues, preserving hexagonal shape even at high temperatures, which has a strong impact on the optical quality and excitonic FSS. The highly symmetric dots obtained with our modified recipe show a mean line width of the neutral exciton of about 15 *μ*eV and a best value of 9 *μ*eV, a mean fine structure splitting of 4.5 *μ*eV, which results in the aforementioned large fraction (more than 95%) of emitters capable of generating entangled photon, as reported in^22^.

## Experimental Details

The growth experiments were performed in a conventional Gen II MBE system, on epiready GaAs (111)A substrates. The optimal control of the As flux during the growth was assured by a valved cell. The cracking zone temperature of the As cell was set in every experiment at 600 °C in order to provide As_4_ molecules.

After the oxide desorption at 580 °C, an atomically smooth surface was prepared by growing a 100 nm thick GaAs buffer layer and a 50 nm Al_0.3_Ga_0.7_As barrier layer after reducing the temperature to 520 °C. To achieve a smooth surface with minimal surface roughness (RMS below 0.5 nm), growth conditions were kept according to^29^. The RHEED pattern clearly showed a (2 × 2) surface reconstruction^30^.

The substrate temperature was then decreased to 450 °C and the As valve closed in order to deplete the growth chamber from the arsenic molecules. When the background pressure reached a value below 1 × 10^−9^ Torr, a Ga flux with a rate of 0.01 ML/s was supplied to the substrate surface to form Ga droplets. During the Ga supply the surface reconstruction did not show any change. One sample with Ga droplets, from now on D, was then removed from the growth chamber.

For the other samples, in order to study the influence of substrate temperature and As flux during the crystallization on the QD formation, the substrate temperature was decreased to low temperature, 200 °C (sample L1) or medium temperature, 400 °C (sample M1) or increased to high temperature, 500 °C (all the samples of H series) and then the Ga droplets irradiated with As flux for 5 minutes. The irradiated As beam equivalent pressure (BEP) is reported in Table 1 with all the parameters used for the QD formation.

**Table 1:** Substrate temperature and Ga flux of fabricated samples for the droplet formation, substrate temperature and As flux for droplet crystallization, final amount of GaAs measured per square micrometer.

Sample	Substrate temp.	Ga amount	Substrate temp.	As BEP	GaAs volume
	during Ga deposition (°C)	MLs	during arsenization (°C)	Torr	(*nm*^3^/*μm*^3^)
D	450	0.4	—	—	—
L1	450	0.4	200	2 × 10^−6^	1.15 × 10^5^
M1	450	0.4	400	2 × 10^−6^	6.2 × 10^4^
H0	450	0.4	500	8 × 10^−7^	8.21 × 10^3^
H1	450	0.4	500	2 × 10^−6^	1.03 × 10^4^
H2	450	0.4	500	5 × 10^−6^	2.07 × 10^4^
H3	450	0.4	500	2 × 10^−5^	3.4 × 10^4^
H4	450	0.4	500	5 × 10^−5^	5.6 × 10^4^
H5	450	0.4	500	7 × 10^−5^	5.72 × 10^4^

A second set of samples was then prepared using the same recipe of the first set, but capping the nanostructures with a 10 nm of AlGaAs barrier layer grown at 500 °C, another 40 nm at 520 °C and a GaAs capping layer of 5 nm. This second set will be recognized adding a C at the end of the sample name.

The morphological characterization of the uncapped samples was performed *ex–situ* by an Atomic Force Microscope (AFM) in tapping mode, using ultra-sharp tips capable of a resolution of about 2 nm. Ensemble photoluminescence measurements (PL) were carried out cooling the samples at 15 K and using the 532 nm line of a Nd:YAG continuous wave laser. The incident power on the samples was 0.5 mW with a laser spot size of approximately 80 *μ*m.

## Results

**Figure 1:**
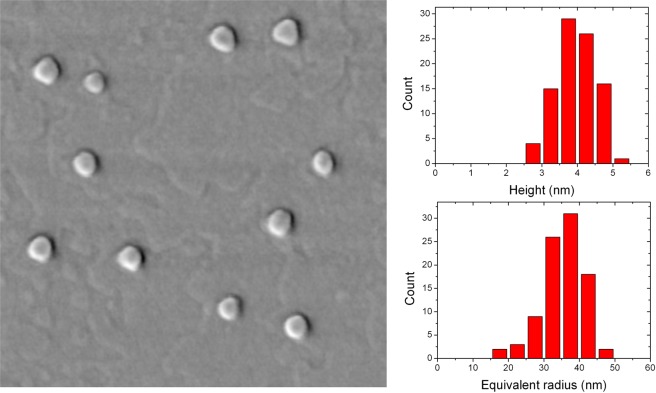
Left panel: 1 × 1 *μ*m^2^ AFM scans of sample H4. Upper and lower right panels: height and equivalend radius distribution for the nanostructures of sample H4.

The AFM characterization of all the uncapped samples was performed by collecting different images in different areas of the sample, in order to study the density, the shape, the volume and the size distribution of the QD ensemble. Left panel of Fig. 1 shows a 1 × 1 *μ*m^2^ AFM scan of sample H4. The image demonstrates the formation of GaAs QDs. The density of QDs calculated over different images on this sample is approximately 8 × 10^8^ cm^−2^ The height and equivalent radius distributions of the QDs are reported in the two right panels of Fig. 1. We found a mean height of 3.9 ± 0.5 nm and a mean equivalent radius of 35.3 ± 5.3 nm.

**Figure 2:**
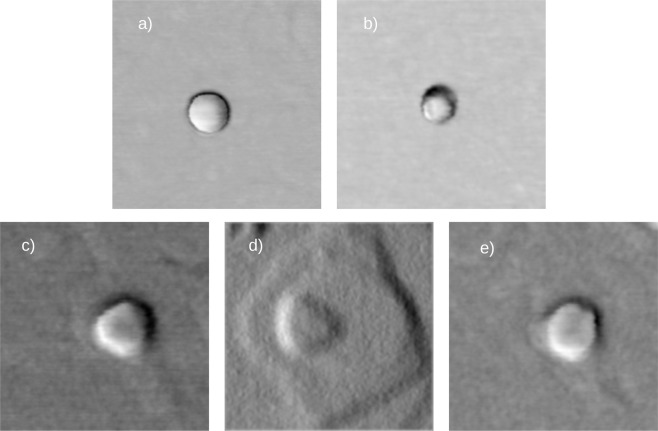
250 × 250 nm^2^ AFM scans of single nanostructure on sample D (panel a), L1 (panel b), M1 (panel c), H1 (panel d) and H4 (panel e).

The AFM characterization of sample D, on which only Ga deposition was performed, shows the formation of Ga droplets with spherical cup shape (see panel a of Fig. 2). The density of the droplets on sample D is approximately 7 × 10^8^ cm^−2^, the diameter is 50.4 ± 7.0 nm and the height 7.4 ± 1.1 nm, the contact angle is approximately 33.7°. The shape of the droplet is perfectly symmetric, without elongation in any crystallographic direction. Simple calculations considering the volume of the droplets, the density, and the amount of the deposited Ga, demonstrate with good agreement that all the gallium is collected inside the droplets. This is in agreement with the fact that (111)A surface is Ga terminated and the Ga excess, during gallium deposition, immediately creates droplets on the surface.

**Figure 3:**
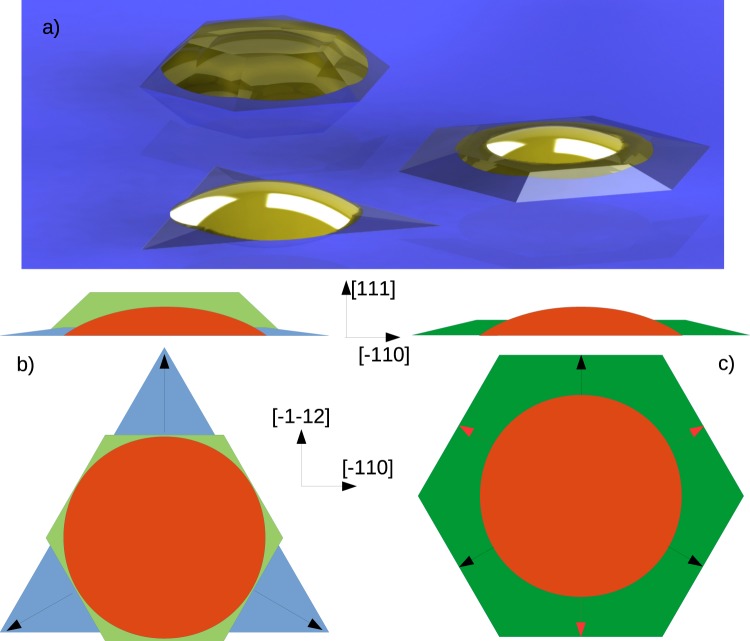
Panel a: sketch of mean sized quantum dot on sample L1, H1 and H4 (in brown, respectively upper, lower left and lower right) compared with the original gallium droplet (yellow). Panel b: graphical representation of mean sized droplet on sample D (orange) and of quantum dot on samples H1 (light blue) and L1 (light green). Panel c: graphical representation of mean sized droplet on sample D (orange) and of quantum dot on samples H4 (dark green). Black and red arrows in panel b and c indicate directions perpendicular to step A and B, respectively.

**Figure 4:**
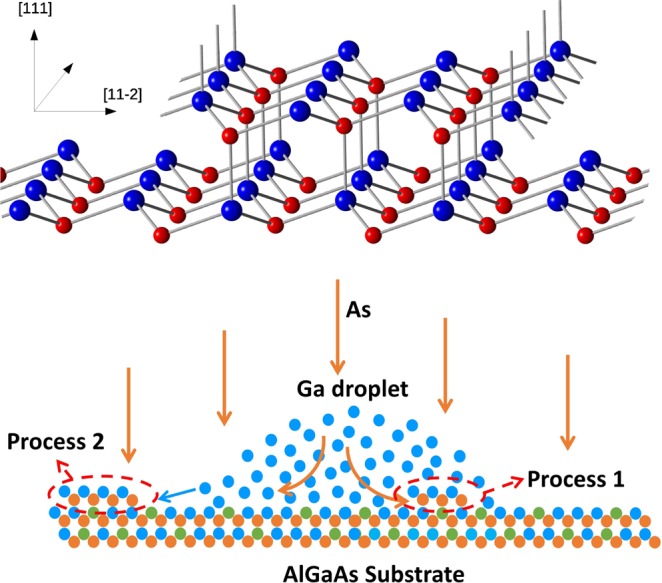
Upper panel: schematic drawing of GaAs (111)A surface. It is possible to notice that Ga atoms (blue spheres) sticking on (11$\bar{2}$) step have three dangling bonds, while Ga atoms sticking on ($\bar{1}\bar{1}$2) step have only two dangling bonds. Lower panel: schematics of the growth processes active during DE. Process 1 refers to As incorporation at the bottom of the droplet. Process 2 refers to Ga atom detachment from the droplet and subsequent incorporation into the crystal via As reaction.

The AFM characterization of samples L1, M1 and H1 (Fig. 2, panel b, c and d respectively), on which droplets were arsenized with a BEP of As of 2 × 10^−6^ Torr at substrate temperature of 200, 400 and 500 °C respectively, shows the formation of QDs with different shapes. On sample L1 (see panel b of Fig. 2) truncated pyramids with regular hexagonal base are formed, while truncated pyramids with equilateral triangular base are formed on H1 sample (see panel d of Fig. 2). The QDs are limited by sides split up into two groups: A sides, facing the $[\bar{1}\bar{1}2]$, $[\bar{1}2\bar{1}]$ and $[2\bar{1}\bar{1}]$ directions, and B sides, facing the $[11\bar{2}]$, $[1\bar{2}1]$, and $[2\bar{1}1]$ directions. These shapes are in agreement with the ones reported for larger islands by Jo *et al*.^15^. On sample H1 the truncated pyramids show a mean height of 2.0 ± 0.4 nm and the equilateral triangle a mean side of 82.1 ± 8.6 nm, while on sample L1 the truncated pyramids show a mean height of 10.8 ± 2.7 nm and the hexagon a mean side of 29.6 ± 3.3 nm. The angle between the substrate and the sidewalls is approximately 35° for sample L1 and 7° for sample H1. Sample M1 (see panel c of Fig. 2) shows an intermediate behavior. The QDs show a truncated pyramidal shape with an irregular hexagonal base. In this case, three sides are longer than the other three (40.1 ± 4.2 and 23.8 ± 3.0 nm, respectively), the mean height 3.0 ± 0.5 nm and the angle between the substrate and the sidewalls is approximately 18°.

Panel e of Fig. 2 shows AFM scan of single QD grown on sample H4, where the droplets were arsenized at 500 °C with a BEP of 5 × 10^−5^ torr. Here the QDs show a truncated pyramidal shape with regular hexagonal base. The mean height is 3.9 ± 0.5 nm, the hexagon side of 42.7 ± 3.2 nm, and the angle between the substrate and the sidewalls is approximately 14°.

Likewise GaAs/AlGaAs droplet epitaxy QDs grown on (001), we expect the dots laterally limited by stepped facets^31^.

**Figure 5:**
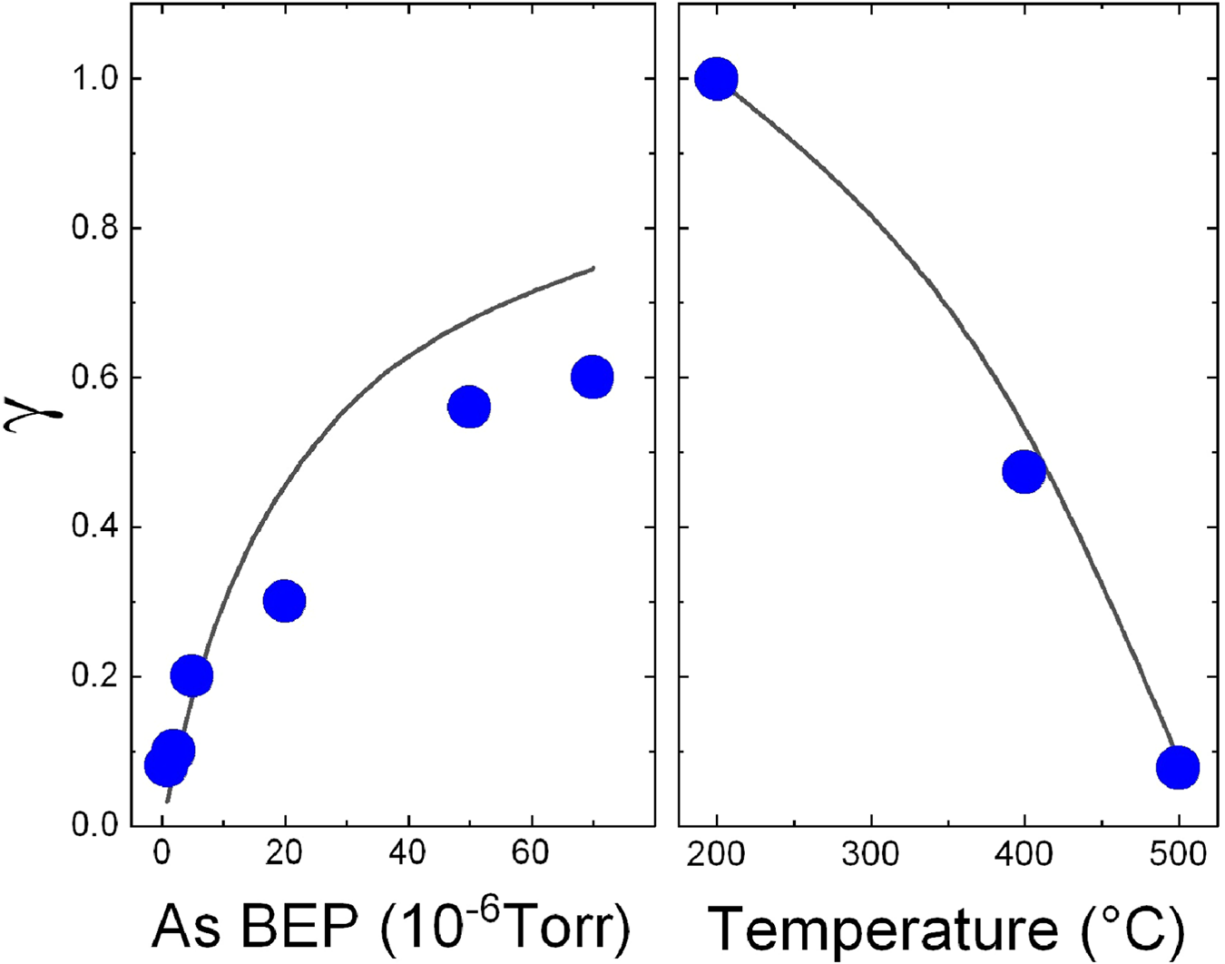
Dependence of the ratio *γ* = *V*_1_/*V* on the As BEP *J*_As_ (left panel) and substrate temperature *T* (right panel). The experimental data are indicated by the red circles. The continuous line reports the fit of the data using Eq. (3).

In order to study the role of Ga adatom diffusion and incorporation during droplet arsenization, we analyzed the AFM images and measured the total volume of GaAs crystallized inside the QDs after the arsenization, as reported in Table 1. An important parameter of the DE–QDs which helps to elucidate the actual processes during the droplet crystallization is the ratio *γ* = *V*_1_/*V* between the volume of the final QDs (*V*_1_) and the GaAs volume available (*V*) by the complete crystallization of the Ga contained in the droplets. It quantitatively sets the difference between a two–dimensional growth, where the droplet have the role of local group III reservoirs^32^ and *γ* = 0, and the three–dimensional crystallization of the QD inside the original droplet. The experimental dependence of *γ* on As flux (*J*_As_) and crystallization temperature *T* is reported in Fig. 5. The measured GaAs crystallized inside the QDs is always lower from the expected volume considering the initial Ga volume stored in the droplets, except for sample L1. The total volume of GaAs crystallized inside the QDs decreases with increasing arsenization temperature and with decreasing *J*_As_.

**Figure 6:**
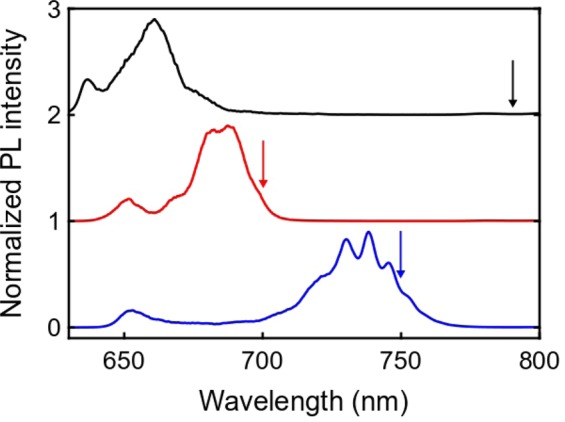
Normalized low-temperature ensemble PL spectra of the samples L1C, H1C and H4C (from top down) capped with a barrier layer. The arrows mark the emission wavelength calculated using realistic dot shapes from AFM images taken from the corresponding uncapped samples and linear dimensions given by average values from the experimental size distribution.

Another important fabrication step, which affects the optical properties of the QDs, is the capping procedure. We used ensemble PL to investigate samples L1C, H1C and H4C as reported in Fig. 6 in black, red and blue, respectively. The peak around either 640 or 650 nm is related to AlGaAs barrier, whereas the emission bands at 650–690, 660–705 and 700–765 nm, for samples L1C, H1C and H4C respectively, are related to QD emission.

## Discussion

The morphology observed for the QDs grown on the sample on which Ga droplets were arsenized with low As flux, L1, M1 and H1, is in agreement with the description reported in^15^, attribuing the morphological evolution to the higher incorporation rate of Ga at A steps, respect to that at the B steps. The observed shape dependence on growth parameters is related to the kinetically controlled growth mode of the droplet epitaxy QDs, where diffusion and incorporation processes play a fundamental role. In particular, on (111)A substrates the QD shape is determined by the interplay between the Ga diffusion along the steps and the different incorporation probability of Ga at A and B step edges^33,34^. Ga atoms are, in fact, more easily incorporated along A steps, where each edge As atom displays two dangling bonds, than on along step B, where each edge As atom has only one dangling bond (see upper panel of Fig. 4), thus reducing the reactivity of step B respect to A.

Let us analyze more in the details the change of the QD morphology as a function of the growth parameters. The shape of the QDs on samples L1 and H1 are graphically summarized in Fig. 3 and compared with the size of the original droplet. Comparing in panel b) the mean dimensions of Ga droplets on sample D (orange), and of QDs on samples L1 (light green) and H1 (light blue), it is possible to see that the formation of QD crystallized with 2 × 10^−6^ torr As BEP, while increasing the substrate temperature from 200 to 500 °C, is dominated by incorporation exclusively along A steps, while the incorporation along B steps is suppressed. It can be observed clearly from panel b of Fig. 3 that the sides of the hexagonal dot on sample L1 (in light green) is mostly tangent to the base circle of the original droplet (orange), and that the sides of triangular dots on sample H1 (light blue) are mostly tangent to the base circle of the original droplet only along B steps, thus confirming the absence of incorporation in those directions. Also on sample M1 (not shown in the picture) the longer sides are mostly tangent along B steps to the base circle of the original droplet.

These observations confirm that the shape of the QDs is determined by kinetically controlled diffusion and incorporation processes in which the different growth velocity between A and B steps determines anisotropy at high temperature.

By increasing the As BEP up to 5 × 10^−5^ torr at high substrate temperature, it is possible to obtain again QDs with hexagonal symmetrical shape. A graphical representation is reported in panel c of Fig. 3. Here we consider the shape and the mean size measured for the original gallium droplet (orange) and we compared it with the shape and the mean size measured for the QDs on sample H4 (dark green). From panel c can also be observed that on sample H4 all the sides of the hexagonal dots are away but at the same distance from the base circle of the original droplet.

Let us discuss the presented phenomenology. We analyze the Ga diffusion length on B steps ${\ell }_{B}$. When ${\ell }_{B} > {L}_{B}$, being *L*_*B*_ the B step length, the Ga atom arriving at B-steps can easily diffuse along the step and then reach the high incorporation rate A steps. The consequent higher growth rate of the A steps, due to both higher incorporation probability and higher Ga atom flux respect to B-Steps, causes the disappearance of the A steps. This leads to a triangular QD shape limited by facets with B steps. On the other side, when ${\ell }_{B} < {L}_{B}$, there is no transfer of Ga atoms between B and A steps and Ga is incorporated at the arrival step. This condition leads to QDs with an hexagonal shape. The ${\ell }_{B}$ is controlled by the Ga diffusivity along the B steps ${D}_{B}\propto \exp (-{E}_{D}kT)$, where *E*_*D*_ is the diffusivity activation energy, and by Ga average time $\tau \propto 1/{J}_{As}$ between the arrival and the reaction at the step: ${\ell }_{B}=\sqrt{{D}_{B}\tau }\propto \exp (-{E}_{D}/2kT){J}_{As}^{-12}$. The diffusion length is therefore minimized by low temperatures and/or high As fluxes growth conditions. The activation energy along B steps ${E}_{A}={E}_{D}/2$ has been recently reported in Ref. ^15^, ${E}_{A}=0.16\,eV$. Comparing samples L1 and H4, the two which exhibit QDs with hexagonal shape, we observe that the the increase in the arsenization temperature from 200 °C (L1) to 500 °C (H4) determines an increase of a factor 5 of the diffusivity term in ${\ell }_{B}$ while the concurrent increase in *J*_As_ from 2 × 10^−6^ Torr (L1) to 7 × 10^−5^ Torr decreases the Ga lifetime around factor 6. Therefore, the two effects cancel each-other in the determination of diffusion length, leaving almost unchanged ${\ell }_{B}$. We then interpret the observed QD hexagonal shape in L1 and H4 samples as the results of ${\ell }_{B} < {L}_{B}$ due to the low temperature (L1) and high As flux (H4) growth conditions. As a result, we can state that Ga diffusion length on B steps is the physical parameter that controls the kinetics of the growth, and thus, in turn, the QD shape.

The described behavior allows for the growth of GaAs QDs by DE at substrate temperature much higher than the one typically used on (001) substrates and, compared to the data previously reported on (111)A substrates, to preserve the hexagonal shape also for arsenization performed up to 500 °C. This is expected to allow for the growth of materials with improved crystalline quality respect to the usual DE QDs crystallized at 200 °C. In fact, a low temperature of crystallization for the Ga droplets^35,36^ and subsequent AlGaAs barrier deposition^37^ is detrimental for the crystalline and the optical quality of the QDs, mainly due to the formation of vacancies and the incorporation of defects.

We then consider the loss of material observed during the crystallization process, as reported in the last column of Table 1. To explain this behavior, we have to consider that the crystallization process involves the control of the growth kinetic, which allows to tune the fabricated nanostructures from three to two–dimensional. On GaAs (001) substrates, nanostructure shape can vary from compact islands to rings and eventually to flat disks extending to the droplet surroundings^38^. These different shapes can be achieved by tuning the speed of the arsenization processes that takes place within the metallic droplet (process 1 in Fig. 4) lower panel, and the Ga atoms diffusion and incorporation outside the droplet (process 2 in Fig. 4 lower panel).

On GaAs (111)A substrates, the geometry of the fabricated nanostructure is different due to different symmetry, and we observe compact islands for all the measured parameter range but we observe that the ratio of Ga crystallized inside the rim of the original droplet and the one outside, is changing with the growth parameters *T* and *J*_As_.

The parameter which quantifies the balance between process 1 (the crystallization inside the original rim of the droplet) and process 2 (diffusion/incorporation) is the ratio *γ*, as it is expected to range from one in the pure three–dimensional growth to zero in the diffusion/incorporation two–dimensional growth. As previously reported for (001) surface, *γ* is a function of *T* and *J*_As_^39^.

In Fig. 5 we reported the data related to parameter *γ* for the temperature series (L1, M1 and H1, from the top down in right panel) and for the As pressure series (from H0 to H5, from left to right, left panel). Considering the temperature series, it is possible to see that on sample L1 almost all the gallium is crystallized inside the hexagonal QDs, while on samples M1 and H1 a loss of about 46% and 91% of the original gallium deposited is measured. Considering the samples of As pressure series, arsenized at 500 °C with different As fluxes from 8 × 10^−7^ to 7 × 10^−5^ Torr, it is also consistently observed a loss in volume. The value of *γ* is increasing with the equivalent pressure of As irradiated during the droplet crystallization. For this series the loss in volume of GaAs crystallized inside the QDs is between 50% and 93%. It is interesting to notice that, on (111)A surface, the possibility of a loss of material outside the final nanostructure was already observed for InAs QDs grown by DE in^40^.

To understand the observed behavior we have to consider how the droplet arsenization and the diffusion processes depend on the growth parameters. Let us first assume the droplet crystallization mechanism depends on the liquid–solid interface area, the arsenic solubility and diffusivity into the droplet and *J*_As_. Considering, as first order approximation, a slow dependence of the interface area on the growth time (process 1 in Fig. 4, the volume crystallized inside the droplet depends linearly on growth time *t* via the equation

1 $${V}_{1}(t)={\rho }_{D}{J}_{{\rm{As}}}t$$

where *ρ*_*D*_ is the constant that takes into account all the other factors (the arsenic solubility and diffusivity into the droplet) with the exception of the As flux *J*_As_.

The growth rate of Ga adatoms incorporated outside the nanostructure depends in a more complex way on *J*_As_ and *T*. The easiest resulting geometry observed on GaAs(001) substrates is a disk, with a radius given by the sum of the diffusion length of Ga atoms (${\ell }_{surf}$) and of the radius of the droplet: $R={\ell }_{surf}({J}_{{\rm{A}}s},T)+{r}_{0}$^39^. Here ${\ell }_{surf}^{2}={D}_{0}\exp (\,-{E}_{D}/kT)({N}_{d}/{J}_{{\rm{A}}s})$, where *D*_0_ is the diffusivity prefactor, *E*_*D*_ the surface diffusion activation energy and *N*_*d*_ the surface density of As sites. The disk is therefore increasing its radius by increasing the substrate temperature and decreasing the As flux. The disk increases its thickness with a rate which is proportional to *J*_As_ and to the product between As/Ga reaction probability and As residence time ${\zeta }_{R}$^41^. Considering that in most of the cases ${\ell }_{surf}\gg {r}_{0}$ (with the exception of the low T range) and that the diffusion/incorporation process follows the same physics on (001) an (111)A, the growth rate of process 2 can be expressed by

2 $$\begin{array}{ccc}{V}_{2}(t) & = & \mu {\prime} {\zeta }_{R}{\ell }_{surf}^{2}{J}_{As}t=\mu {\zeta }_{R}[{D}_{0}\exp (\,-{E}_{D}/kT){J}_{As}^{-1}]{J}_{As}t\\  & = & \mu {\zeta }_{R}{D}_{0}\exp (\,-{E}_{D}/kT)t\end{array}$$

Where *μ*′ and *μ* are constant collecting geometrical and constant factors. The growth will proceed for a time *τ* until the Ga in the droplet is fully consumed. This condition is reached when $V={V}_{1}(\tau )+{V}_{2}(\tau )$. Combining Eq. (2) equation with Eq. (1)

3 $$\gamma ({J}_{{\rm{As}}},T)={\left[1+\frac{\mu {\zeta }_{R}{D}_{0}\exp (-{E}_{D}/kT)}{{\rho }_{D}{J}_{{\rm{As}}}}\right]}^{-1}$$

It is possible to observe that in Eq. 3, *γ* is increased by increasing the As flux *J*_As_ as *γ* = *J*_*As*_/(*J*_*As*_ + *const*), and is decreased by increasing *T* as $\gamma ={[1+const\exp (-{E}_{D}/{k}_{B}T)]}^{-1}$. Also increasing the term $\mu {\zeta }_{R}{D}_{0}/{\rho }_{D}$, the value of *γ* is decreased. For this reason, a short As residence time ${\zeta }_{R}$ on (111)A respect to (001) substrates, as reported in^42^, is expected to make the contribution of the crystallization process inside the droplet to be preminent even at high T, provided a sufficient increase of the As BEP. The upper limit for this effect is marked by the limited As solubility and diffusivity in the droplet for extremely high As flux.

The dependence of *γ* on *J*_As_ and *T* is reported graphically in Fig. 5 and compared with experimental values of the ratio. For the diffusion activation energy on GaAs (111)A surface we used the value as calculated by ref. ^43^, *E*_*D*_ = 1.06 eV. The value of the ratio $\mu {\zeta }_{R}{D}_{0}/{\rho }_{D}=2\times {10}^{2}$ Torr has been fitted to the temperature series L1, M1, and H1. The data are nicely reproduced by our model which depends on a single fit parameter.

Understanding the relationship between the growth parameters and the shape of the nanostructures is fundamental for the fabrication of emitters with specific electronic and optical properties but it is then necessary to evaluate the effect of the deposition of the AlGaAs capping layer in terms of shape change and interdiffusion. The optical properties of the QDs capped with 50 nm Al_0.3_Ga_0.7_As and 5 nm of GaAs were studied by means of ensemble photoluminescence (PL) and single-band constant-potential model simulations on the second set of sample. As expected from simple considerations of quantum confinement energy, the size of the QDs, and in particular their height, is affecting the emission wavelength. The ensemble PL spectra of samples L1C, H1C and H4C are displayed in black, red and blue, respectively, in Fig. 6. The peak around either 640 or 650 nm is related to AlGaAs barrier, whereas the emission bands at 650–690, 660–705 and 700–765 nm, for samples L1C, H1C and H4C respectively, are related to QD emission. The different position of AlGaAs related peak in sample L1C can be attributed to a slightly different composition in the barrier. The distribution of the emission energy for sample H4C shows sizable modulations which are typical of QDs with low aspect ratio and can be attributed to monolayer fluctuations in height, as reported in^24^.

We simulated the expected radiative recombination energies with the single–band constant-potential model^44^, using realistic dot shapes from the AFM images taken from the corresponding uncapped samples and linear dimensions given by average values from the experimental size distribution. The band parameters used in the calculation^45,46^ are chosen consistently with previous studies on droplet epitaxy GaAs/AlGaAs QDs. The results for the ground state transition are shown by arrows in Fig. 6 alongside the ensemble PL spectra. It is worth notice that for the samples in which the substrate temperature during the QDs crystallization was set equal or higher than 400 °C, a small blueshift around 30 meV was found between the theoretical estimation and the centroid of the energy distribution. For a substrate temperature of 200 °C during the As crystallization (sample L1C), a blueshift larger than 200 meV was measured. We interpret such discrepancy as the outcome of interdiffusion at the AlGaAs/GaAs interface during the capping layer deposition, when the temperature is substantially increased up to 500 °C and the nanostructure is slowly covered with an AlGaAs layer. While the interdiffusion processes during the deposition of the capping layer are always present, they have much more limited impact for QDs crystallized at elevated temperature, and can be estimated in an interdiffusion of few monolayers as shown by cross section scanning tunneling microscpy measurements on DE-QDs^47^. In fact, when the QDs are crystallized at lower temperature, we expect the presence of a larger amount of vacancies and crystal defect, which enhance the interdiffusion process (see e.g.^35,36^) when the temperature is raised again to 500 °C during the AlGaAs barrier deposition. According to previous estimates of the interdiffusion length in DE QDs^35,36^, we expect and interdiffusion lenght L ≥ 1 nm for the QDs grown at low temperature, thus being a sizeable fraction of the total QD height.

As shown in^22^, micro–PL measurement of sample H4C confirms the high quality of the QDs. The measurements performed on the sample show a mean line width of the neutral exciton of about 15 *μ*eV and a best value of 9 *μ*eV, a mean fine structure splitting of 4.5 *μ*eV, which results in the aforementioned large fraction (more than 95%) of emitters capable of generating entangled photons. The higher crystallization temperature of the Ga droplets allows for the formation of nanostructures with a reduced density of vacancies and defects. The possibility to set a higher substrate temperature without introducing anisotropy and then FSS, is made possible by the short residence time of As on (111)A respect to (001) substrates. A highly symmetric hexagonal shape also at high crystallization temperature is obtained controlling the Ga diffusion along B steps by increasing As flux, which permits to properly balance the Ga adatom incorporation along steps A and B.

## Conclusions

The presented high temperature droplet epitaxy procedure allows for the self–assembly of QDs with high optical quality and symmetric shape by crystallizing the QDs at substrate temperature higher than previously reported^15^. The improved optical quality is related to the high crystalline quality of the GaAs QDs and the surrounding AlGaAs barrier, as both are crystallized or deposited at a temperature close to the optimal one for the GaAs crystal growth on (111)A surface. We investigated and modeled the dependence of the QD shape and size on the parameters used during the crystallization process. The model proposed shows that high temperature droplet epitaxy on (111)A substrates is governed, as the standard droplet epitaxy on GaAs(001), by the balance between crystallization within the droplet and the process of Ga adatom detachment from the droplet, diffusion and incorporation into the crystal surrounding the droplet. The predominance of the former over the latter allows for the self–assembly of 3D islands. This is realized on GaAs (111)A substrates at high T owing to the low residence time of the As on the (111)A surface which hinders the diffusion/crystallization processes on the crystal surface around the droplet. The high As pressure required for the crystallization also permits the equalization of the growth velocities along the A and B steps by increasing the incorporation of Ga adatoms along the B steps, resulting in a symmetric hexagonal shape. PL measurements also demonstrated that high temperature of crystallization permits to preserve the shape of the QDs when capped, thus allowing for the reproducibility of the fabrication procedures which is a fundamental asset for the deterministic design of the emitters for wavelength–specific applications.
